# Risk factors of bloodstream infection after allogeneic hematopoietic cell transplantation in children/adolescent and young adults

**DOI:** 10.1371/journal.pone.0308395

**Published:** 2024-08-07

**Authors:** Daichi Sajiki, Hideki Muramatsu, Manabu Wakamatsu, Daiki Yamashita, Ryo Maemura, Yusuke Tsumura, Masayuki Imaya, Ayako Yamamori, Kotaro Narita, Shinsuke Kataoka, Rieko Taniguchi, Atsushi Narita, Nobuhiro Nishio, Yoshiyuki Takahashi

**Affiliations:** 1 Department of Pediatrics, Nagoya University Graduate School of Medicine, Nagoya, Aichi, Japan; 2 Center for Advanced Medicine and Clinical Research, Nagoya University Hospital, Nagoya, Aichi, Japan; University of South Carolina School of Medicine, UNITED STATES OF AMERICA

## Abstract

Allogeneic hematopoietic cell transplantation (HCT) is a crucial treatment for various diseases, including hematological malignancies, solid tumors, and genetic disorders. Despite its curative potential, HCT is associated with severe complications, notably infections, graft-versus-host disease, and organ damage. Infections, particularly bloodstream infections (BSIs), pose a significant threat in the initial weeks post-HCT, necessitating effective management strategies. This retrospective study aimed to clarify the incidence, pathogens, and risk factors associated with BSI within the first 30 days after allogeneic HCT in children/adolescents and young adults (AYAs). The study included 115 patients aged <31 years who underwent 121 allogeneic HCTs at the Department of Pediatrics, Nagoya University Hospital between January 1, 2018, and March 31, 2022. Data encompassed demographic characteristics, HCT details, and BSI information. Overall, 27 of 121 patients developed BSI with the cumulative incidence of 23.5% (95% confidence intervals [CI]: 17.0%–30.6%) at 30 days after HCT. The median onset time of BSI was 7 (range, 4–26 days) after HCT. Gram-positive bacteria accounted for 89% of pathogens isolated from blood cultures, with *Streptococcus mitis/oralis* being the most common. In multivariable analysis, tandem HCT (subdistribution hazard ratio [SHR]: 5.67, 95% CI: 2.74–11.7, *p *< 0.001) and peripherally inserted central catheters (SHR: 2.96, 95% CI: 1.34–6.55, *p*  =  0.007) were identified as independent risk factors for BSI. In patients receiving tandem HCT, the pathogens isolated from blood cultures were all gram-positive bacteria, with *Streptococcus mitis/oralis* accounting for up to 67% of the isolated pathogens. Tandem HCT and PICCs were identified as independent risk factors for BSI after allogeneic HCT in children/AYAs. The pathogens were commonly gram-positive, and *Streptococcus mitis/oralis* is important in patients who received tandem HCT. These data can provide valuable information for future studies to consider effective interventions to reduce the risk of BSI in high-risk patients.

## Introduction

Allogeneic hematopoietic cell transplantation (HCT) has been used to treat various diseases, including hematological malignancies, solid tumors, and genetic diseases [1]. Although HCT can cure these patients, it often leads to life-threatening side effects such as infections, graft-versus-host disease, and organ damage. Infections is the most common serious complication that develops within the first few weeks after HCT.

Patients are treated with immunosuppressive drugs to support the engraftment of the donor’s hematopoietic stem cells to the recipient, which leads to immune system suppression. Neutrophils, which play an important role in fighting against bacterial and fungal infections, are present in undetectable levels in the first few weeks after HCT until neutrophils are derived from the transplanted donor’s hematopoietic stem cells. During this period, patients are highly susceptible to bacterial and fungal infections. Even after hematopoietic stem cells are engrafted and differentiated into various hematopoietic cells, patients are still susceptible to infection. In this phase, patients are more susceptible to viral or fungal infection than to bacterial infection, because the functions of lymphocytes are diminished owing to the use of immunosuppressive drugs to prevent graft-versus-host disease.

Bacterial and fungal infections can occur in any organ. Bloodstream infection (BSI), in which bacteria or other microorganisms enter the bloodstream and spread throughout the body, is a severe form of infection. It causes organ damage and occasionally leads to sepsis. The incidence of BSI after HCT ranges from 8.7% to 34% [2, 3], and the majority of events occurred in the pre-engraftment phase [4–6]. BSI after HCT is associated with high mortality rates [7, 8], and it should be managed to perform a safe and effective HCT. The risk factors for BSI reported to date include the use of unrelated donors, umbilical cord blood, and myeloablative conditioning regimens, mucositis, and high-risk malignant diseases [2, 9–12]. However, the incidence, risk factors, and associated pathogens of BSI vary widely by geographic location and patient population [2]. To effectively manage BSI after HCT, we retrospectively investigated the responsible pathogens and risk factors of BSI after HCT in children/adolescents and young adults (AYAs) at our hospital.

## Materials and methods

### Participants

This study included children/AYAs (< 31 years old) who received allogeneic HCT at the Department of Pediatrics, Nagoya University Hospital between January 1, 2018 and March 31, 2022.

This study was approved by the ethics committee of Nagoya University Graduate School of Medicine (approval no. 2022–0276). An opt-out consent process was approved by disseminating the research information; thus, the requirement of informed consent was waived. All research processes were conducted in accordance with the principles of the Declaration of Helsinki.

### Data collection

Medical records of the patients were reviewed to retrieve data on the demographic characteristics of the participants; HCT details such as donor source, patient and donor human leukocyte antigen, conditioning regimen, supportive care, catheter insertion, and adverse events evaluated using the Common Terminology Criteria for Adverse Events v5.0; and information on BSI including onset time, isolated pathogens, and treatment outcomes.

Data were collected between October 24, 2022 and April 30, 2023. All personal data were coded to ensure that individuals could not be identified in the analysis.

### Statistical analysis

The cumulative incidence of BSI was calculated using the competing risk method and compared using Gray’s test. The subdistribution hazard ratios of BSI development were obtained using the Fine–Gray proportional hazards model. All tests were two-tailed. A type I error of <0.05 was considered statistically significant. All statistical analyses were performed using EZR (Saitama Medical Center, Jichi Medical University, Saitama, Japan) [13].

### Definition

A definitive BSI was defined as the isolation of a pathogen from the blood of a patient with fever or other signs of infection. For common skin contaminants such as coagulase-negative staphylococci and *Corynebacteria*, BSI was confirmed only after two consecutive blood cultures were positive for the same bacterial strain [14]. We also defined a probable BSI as a case in which only one set of blood cultures was obtained, common skin contaminants were detected in the blood culture within a short period (≤72 hours), and the clinical picture was considered highly consistent with BSI. The current study aimed to analyze BSI that occurred within 30 days after allogeneic HCT. Conditioning regimens used before transplantation were classified as myeloablative and reduced-intensity regimens, as described by Bacigalupo *et al*. [15]. Tandem HCT was defined as a planned allogeneic HCT after autologous HCT with high-dose chemotherapy provided within 6-month intervals.

## Results

### Characteristics of the patients

We performed 127 allogeneic HCTs for 117 consecutive patients aged <31 years at the Department of Pediatrics, Nagoya University Hospital, between January 1, 2018, and March 31, 2022. After excluding two patients who underwent six HCTs as a rescue treatment for primary graft failure (n = 4) and those who did not receive the conditioning regimen (n = 2), we included 115 consecutive patients who underwent 121 allogeneic HCTs in this retrospective study (**S1 Fig**). **Table 1** and **S1 Table** show the clinical and transplantation characteristics of 115 patients (121 allogeneic HCTs).

**Table 1:** Patient and transplantation characteristics.

	Total cohort	Patients with BSI*	Patients without BSI
	N = 121	n = 27	n = 94
Age at HCT, n (%)			
< 6 years old	54 (45)	12 (44)	42 (45)
≥ 6 years old	67 (55)	15 (56)	52 (55)
Gender, n (%)			
Male	72 (60)	12 (44)	60 (64)
Female	49 (40)	15 (56)	34 (36)
Disease, n (%)			
Benign disease, other than IEI	23 (19)	2 (7)	21 (22)
IEI	19 (16)	3 (11)	16 (17)
Hematological malignancies	35 (29)	2 (7)	33 (35)
Solid tumors	44 (36)	20 (74)	24 (26)
Donor type, n (%)			
Matched related (8/8 allele matched)	13 (11)	0 (0)	13 (14)
Mismatched related (7/8 allele matched)	3 (2)	0 (0)	3 (3)
Matched unrelated (8/8 allele matched)	15 (12)	1 (4)	14 (15)
Mismatched unrelated (7/8 allele matched)	20 (17)	4 (15)	16 (17)
Haploidentical	11 (9)	1 (4)	10 (11)
Cord blood	59 (49)	21 (78)	38 (40)
Conditioning regimen, n (%)			
Myeloablative conditioning	49 (40)	4 (15)	45 (48)
Reduced intensity conditioning	72 (60)	23 (85)	49 (52)
Total body irradiation, n (%)			
None	19 (16)	0 (0)	19 (20)
Low dose (< 8 Gy)	70 (58)	23 (85)	47 (50)
High dose (≥ 8 Gy)	32 (26)	4 (15)	28 (30)
Tandem HCT, n (%)			
No	100 (83)	15 (56)	85 (90)
Yes	21 (17)	12 (44)	9 (10)
Catheter, n (%)			
Tunneled CVC	101 (83)	20 (74)	81 (86)
PICC	18 (15)	7 (26)	11 (12)
Tunneled CVC and PICC	2 (2)	0 (0)	2 (2)
Catheter retention time**, n (%)			
< 45 days	61 (50)	8 (30)	53 (56)
≥ 45 days	60 (50)	19 (70)	41 (44)
Antibiotic use at day 0 of HCT, n (%)			
No	67 (55)	19 (70)	48 (51)
Yes	54 (45)	8 (30)	46 (49)
History of BSI within 6 months prior to HCT, n (%)			
No	106 (88)	24 (89)	82 (87)
Yes	15 (12)	3 (11)	12 (13)
Active infections at the time of HCT, n (%)			
No	115 (95)	26 (96)	89 (95)
Yes	6 (5)	1 (4)	5 (5)
Oral mucositis (CTCAE v5.0)			
< Grade 2	63 (52)	18 (67)	45 (48)
≥ Grade 2	58 (48)	9 (33)	49 (52)

*Definitive BSI (n = 24) and probable BSI (n = 3).

**The number of days from catheter insertion to HCT. Abbreviations: BSI, bloodstream infection; CTCAE, common terminology criteria for adverse events; CVC, central venous catheter; HCT, hematopoietic cell transplantation; HLA, human leukocyte antigen; IEI, inborn errors of immunity; PICC, peripherally inserted central catheter.

### Prophylaxis and treatment of infection

All patients were treated at the hospital for at least 60 days. Polymyxin B sulfate (100,000 units/kg/day), fluconazole (3 mg/kg/day), and valacyclovir (75 mg/kg/day for <10 kg, 50 mg/kg/day for ≥10 kg) were administered until 90 days after HCT to prevent infections. Sulfamethoxazole/trimethoprim (4–8 mg/kg/day as trimethoprim, 3 days/week) was also administered after engraftment and continued for 1 year after HCT. Within the first 90 days after allogeneic HCT, patients received intravenous immunoglobulin (100–200 mg/kg) once a week.

### Development of BSIs

**Fig 1:**
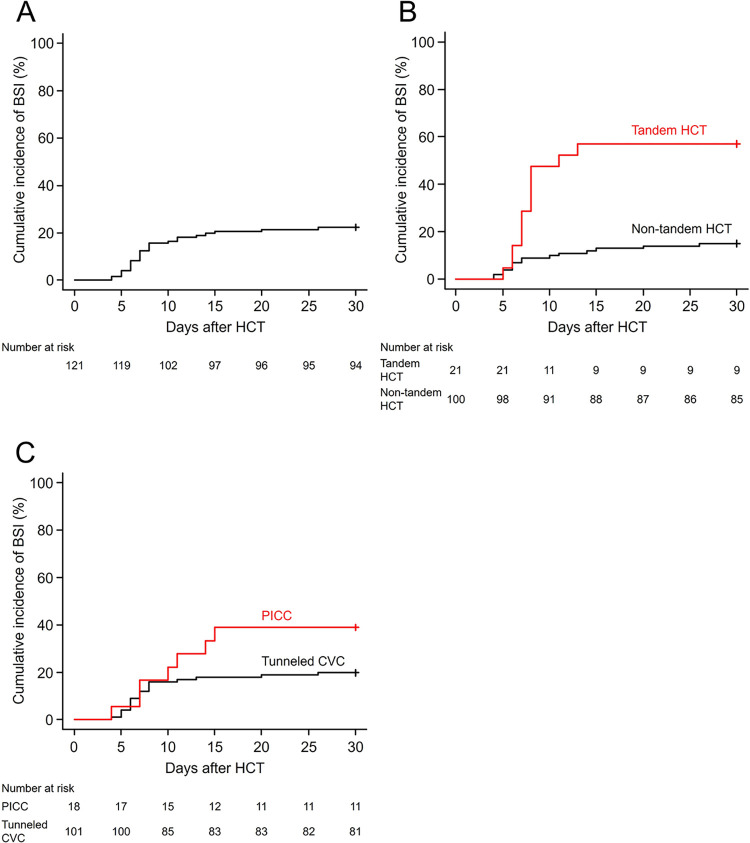
The cumulative incidence of bloodstream infection. (A) The cumulative incidence of bloodstream infection 30 days after HCT in the study cohort was 23.5% (95% confidence intervals [CI]: 17.0%–30.6%). The cumulative incidence of BSI was higher (B) in patients who received tandem HCT than in those who did not (57.1% [95% CI: 32.9%–75.5%] *vs*. 15.0% [95% CI: 8.8%–22.7%], *p* < 0.001) and (C) in patients with PICCs than in those with tunneled CVCs (38.9% [95% CI: 16.8%–60.6%] *vs*. 19.8% [95% CI: 12.7%–28.1%], *p*  =  0.092). Abbreviations: BSI, bloodstream infection; CVCs, central venous catheters; HCT, hematopoietic cell transplantation; PICCs, peripherally inserted central catheters.

**Fig 2:**
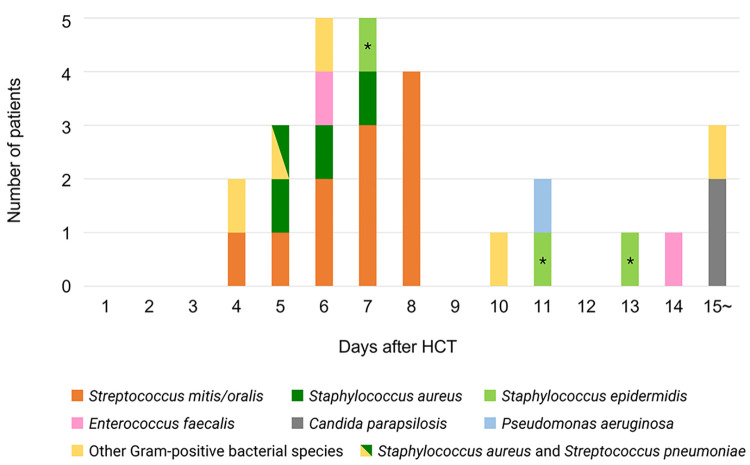
The relationship between pathogens isolated from blood cultures and the onset of bloodstream infection. The number of patients based on the onset of bloodstream infection is indicated by a different color for each pathogen isolated. The asterisk (*) indicates probable BSI patients. Abbreviations: HCT, hematopoietic cell transplantation.

From 0 to 30 days after HCT, 27 of 121 patients developed BSI (definitive BSI [n = 24] and probable BSI [n = 3]) (**Fig 1A**). The median BSI onset was 7 days (range, 4–26 days) after HCT, and all but one patient developed BSI prior to engraftment. **Table 2** and **S2 Table** enlist the bacterial and fungal species found in 27 patients. Gram-positive bacteria were the most common pathogen (*Streptococcus mitis/oralis* [n = 11], followed by *Staphylococcus aureus* [all methicillin-sensitive *Staphylococcus aureus*, n = 4]) (**Table 2** and **Fig 2**). Multidrug-resistant organisms were detected in only one case with multidrug-resistant *Pseudomonas aeruginosa*, for which the patient was a carrier prior to HCT.

**Table 2:** Pathogens isolated from blood culture.

Species		All patients (N = 121)
**Number of blood stream infections, n**		**27**
**Gram-positive bacteria, n (%)**		**25 (89)**
	*Streptococcus mitis/oralis*, n	11
	*Staphylococcus aureus*, n	4*
	*Staphylococcus epidermidis*, n	3**
	*Enterococcus faecalis*, n	2
	*Enterococcus faecium*, n	1
	*Gemella haemolysans*, n	1
	*Staphylococcus haemolyticus*, n	1
	*Streptococcus agalactiae*, n	1
	*Streptococcus pneumoniae*, n	1*
**Gram-negative bacteria, n (%)**		**1 (4)**
	*Pseudomonas aeruginosa*, n	1
**Fungus, n (%)**		**2 (7)**
	*Candida parapsilosis*, n	2

*In one patient, *Staphylococcus aureus* and *Streptococcus pneumoniae* were simultaneously isolated from blood culture in a single blood stream infection episode.

**Probable BSI (n = 3).

Abbreviations: HCT, hematopoietic cell transplantation.

### Univariable and multivariable analyses of risk factors for BSI

The findings of the univariable and multivariable analyses of risk factors for BSI are summarized in **Table 3**. In the univariable analysis, the cumulative incidence of BSI was higher in patients receiving tandem HCT, reduced-intensity conditioning, human leukocyte antigen-mismatched HCT, cord blood transplantation, or having malignant diseases (**Table 3**). In multivariable analysis, tandem HCT (subdistribution hazard ratio [SHR]: 5.67, 95% confidence intervals [CI]: 2.74–11.7, *p *< 0.001) and peripherally inserted central catheters (PICCs; SHR: 2.96, 95% CI: 1.34–6.55, *p*  =  0.007) were identified as independent risk factors for BSI (**Table 3** and **Fig 1B and 1C**).

**Table 3:** Univariable and multivariable analyses of cumulative incidence of blood stream infections.

Variable	Univariable		Multivariable	
	HR (95% CI)	*P*	HR (95% CI)	*P*
Age at HCT				
< 6 years old	1		-	
≥ 6 years old	1.00 (0.48–2.12)	0.990	-	-
Gender				
Male	1		-	
Female	2.09 (1.00–4.39)	0.051	-	-
Disease				
Benign	1		-	
Malignant	2.71 (1.08–6.79)	0.034	-	-
Source				
BM and/or PBSC	1		-	
CB	4.37 (1.81–10.5)	0.001	-	-
HLA (8 allele)				
Matched	1		-	
Mismatched	9.37 (1.30–67.5)	0.026	-	-
Conditioning regimen				
Myeloablative conditioning	1		-	
Reduced intensity conditioning	4.31 (1.48–12.6)	0.008	-	-
Total body irradiation				
< 8 Gy	1		-	
≥ 8 Gy	0.47 (0.16–1.37)	0.170	-	-
Tandem HCT				
No	1		1	
Yes	4.82 (2.31–10.1)	< 0.001	5.67 (2.74–11.7)	< 0.001
Catheter				
Tunneled CVC	1		1	
PICC	2.07 (0.92–4.67)	0.079	2.96 (1.34–6.55)	0.007
Catheter retention time				
< 45 days	1		-	
≥ 45 days	2.79 (1.26–6.19)	0.012	-	
Antibiotic use at day 0 of HCT				
No	1		-	
Yes	0.46 (0.21–1.01)	0.054	-	-
History of BSI within 6 months prior to HCT				
No	1		-	
Yes	0.80 (0.27–2.41)	0.690	-	-
Active infections at the time of HCT				
No	1		-	
Yes	0.69 (0.10–4.67)	0.710	-	-
Oral mucositis (CTCAE v5.0)				
< Grade 2	1		-	
≥ Grade 2	0.62 (0.15–2.61)	0.519	-	-

Abbreviations: BM, bone marrow; BSI, bloodstream infection; CB, cord blood; CI, confidence intervals; CTCAE, common terminology criteria for adverse events; CVC, central venous catheter; HCT, hematopoietic cell transplantation; HLA, human leukocyte antigen; HR, hazard ratio; PBSC, peripheral blood stem cells; PICC, peripherally inserted central catheter.

### Subgroup analysis of BSIs in patients undergoing tandem HCT

**S3 Table** shows the clinical and transplant characteristics of 21 patients who underwent tandem HCT. In total, 12 of 21 patients developed BSI, with a cumulative incidence of BSI at 30 days after HCT of 57.1% (95% CI: 32.9%–75.5%; **Fig 1B**). All pathogens isolated from blood cultures were gram-positive bacteria, with *Streptococcus mitis/oralis*, accounting for 67% (**S2 Table**); 83% of BSI cases developed <10 days after HCT (**S2A Fig**). Univariable analysis did not identify other factors associated with the cumulative incidence of BSI (**S4 Table**).

### Subgroup analysis of patients receiving non-tandem HCT

**S5 Table** depicts the clinical and transplant characteristics of 100 patients who underwent non-tandem HCT. A total of 15 patients developed BSI, with a cumulative incidence of BSI at 30 days after HCT of 15.0% (95% CI: 8.8%–22.7%; **Fig 1B**). Although the bacterial species varied between patients who received tandem and non-tandem HCT, 13 (81%) of 16 pathogens isolated from blood cultures of patients non-tandem HCT were gram-positive organisms (**S2 Table**). BSI developed < 10 days after HCT in 60% cases (**S2B Fig**). Univariable analysis of patients who received non-tandem HCT revealed that patients with PICCs (SHR: 3.51, 95% CI: 1.29–9.58, *p* = 0.014) and female patients (SHR: 3.56, 95% CI: 1.25–10.1, *p* = 0.017) had a higher cumulative incidence of BSI (**S6 Table**).

## Discussion

In this retrospective study, the clinical features and risk factors for BSI in the first 30 days after allogeneic HCT in a cohort of children/AYAs at a single institution were analyzed. From 0 to 30 days after HCT, 27 of 121 patients developed BSI, and tandem HCT and PICCs were identified as independent risk factors for BSI. The use of PICC catheters is increasing with the development of novel devices. In addition, based on relatively recent evidence, tandem HCT has become widely used, especially in pediatric neuroblastoma cases [16, 17]. This is unique because there are no reports that have identified PICC and tandem HCT (a relatively new clinical factor in pediatric HCT) as a possible risk factor for BSI.

It is unclear why tandem HCT was found as a risk factor for early post-HCT BSI in the current cohort. All tandem transplant recipients in this cohort (n = 21) underwent allogeneic cord blood transplant after autologous transplant to improve outcomes in high-risk neuroblastoma, but do not include cases enrolled in ongoing prospective trials (jRCTs041210034). Recent studies have reported that pre-transplant oral/intestinal microbiome profiles are strongly associated with the development of serious infections early after HCT [18, 19]. Another study reported that the diversity of the oral microbiome considerably decreased after HCT; however, it was restored to its pre-HCT composition by 3 months [20, 21]. Future studies should be conducted to assess the impact of microbiome changes on the infectious complications of tandem HCT in patients receiving the initial HCT.

In this study, 89% of the pathogens isolated from patients were gram-positive bacteria, and only one case (4%) had gram-negative bacteria. Gut decontamination with polymyxin B in patients with HCT has been a long-standing institutional practice at Nagoya University Hospital due to its broad gram negative coverage and lack of systemic absorption. This clinical practice may have contributed to a reduction in the rate of gram-negative bacterial infections in this cohort. In addtion, there was also a trend toward a lower incidence of BSI in patients who received intravenous antibiotics on day 0 of HCT (HR 0.46 [0.21–1.01], *p* = 0.054). The routine use of prophylactic antibiotics in pediatric patients undergoing HCT is not recommended by international guidelines [22–24]. Levofloxacin is widely used to prevent bacteremia in adult patients undergoing HCT. However, data supporting the effectiveness of this approach in children are inconsistent [3]. Recently, Gardner et al. [25] reported the effectiveness of levofloxacin in pediatric and adult patients receiving HCT in reducing the incidence of bacteremia. However, it is associated with long-term toxicity to the musculoskeletal system in children [26]. A recent study found that pediatric patients with HCT who received intravenous prophylactic antibiotics (cefepime or piperacillin/tazobactam) had an extremely low incidence of BSI (3%) but a high incidence of *Clostridioides difficile* infection (10%) [27]. Hence, future prospective studies should be performed to evaluate the use of prophylactic antibiotics in specific patient groups at high risk for BSI, such as pediatric patients receiving tandem HCT.

PICC is a non-tunneled central venous catheter (CVC) inserted via the peripheral vein in the upper arm. This catheter is widely used in pediatric patients with hematologic and neoplastic diseases because of its more convenient insertion and removal than the tunneled CVCs. In this study, PICC was considered a risk factor of BSI after allogeneic HCT (**Table 3**), particularly in non-tandem HCT (**S6 Table**). Several studies have investigated the incidence of BSI in pediatric patients with PICCs and tunneled CVCs. Nevertheless, it is unclear whether PICCs are a risk factor for BSI [28–32]. The types of CVC devices appropriate for pediatric patients undergoing HCT should be evaluated in larger patient populations.

There are several limitations of this study. First, this was a single-center, retrospective study of a relatively small number of patients, and the timing and type of antibiotics used were not strictly standardized. In addition, the results should be interpreted with caution because of the relatively small number of events (n = 27) in the overall cohort, which reduces the power of statistical analyses. Second, because this cohort consists of pediatric patients, we have included cases in which the standard two sets of blood cultures were not performed due to difficulties with venipuncture. There were four cases in which common skin contaminants were positive for blood culture (*Staphylococcus epidermidis*: 3, *Staphylococcus haemolyticus*: 1), but only one case in which two sets of blood cultures were obtained and both were positive. However, the remaining three cases had positive blood cultures within a short period of time (72 hours or less), and their clinical presentation indicated a high probability that they were true BSIs, so they were treated as "probable BSIs" in this study. Third, this retrospective analysis precluded a combined analysis of the oral/gut microbiome, which has recently been implicated in post-HCT infectious complications. Future multicenter prospective studies should be conducted to provide a more accurate image of the pathogenesis, biomarkers, and risk factors of BSI in patients who received HCT.

In conclusion, this study identified tandem HCT and PICCs as independent risk factors for BSI in the early phase after allogeneic HCT in children/AYAs. The pathogens were commonly gram-positive, and *Streptococcus mitis/oralis* is important in patients who received tandem HCT. These data can provide important information for future studies investigating the interventions considered effective in reducing the risk of BSI in high-risk patients.

## Supporting information

S1 Fig Transplant cohorts in this retrospective study. There were 127 allogeneic HCTs. Six HCTs were excluded, and the final cohort included 121 HCTs. Abbreviations: HCT, hematopoietic cell transplantation.
(TIF)

S2 Fig The relationship between pathogens isolated from blood cultures and the onset of BSI in patients receiving tandem and non-tandem HCT. The number of patients based on the onset date of bloodstream infection in patients who received (A) tandem HCT and (B) non-tandem HCT is indicated by a different color for each pathogen isolated. The asterisk (*) indicates probable BSI patients. Abbreviations: HCT, hematopoietic cell transplantation.
(TIF)

S1 Table Detailed information about each transplantation in this cohort. (XLSX)

S2 Table Pathogens isolated from blood culture in patients with tandem and non-tandem HCT. (DOCX)

S3 Table Patient and transplantation characteristics of tandem HCT. (DOCX)

S4 Table Univariable analyses of cumulative incidence of blood stream infections in tandem HCT patients. (DOCX)

S5 Table Patient and transplantation characteristics of non-tandem HCT. (DOCX)

S6 Table Univariable analyses of cumulative incidence of blood stream infections in non-tandem HCT patients. (DOCX)

## References

[1] Duarte RF, Labopin M, Bader P, Basak GW, Bonini C, Chabannon C, et al. Indications for haematopoietic stem cell transplantation for haematological diseases, solid tumours and immune disorders: current practice in Europe, 2019. Bone Marrow Transplant. 2019; 54: 1525–1552. doi: 10.1038/s41409-019-0516-2 30953028

[2] Dandoy CE, Ardura MI, Papanicolaou GA, Auletta JJ. Bacterial bloodstream infections in the allogeneic hematopoietic cell transplant patient: new considerations for a persistent nemesis. Bone Marrow Transplant. 2017; 52: 1091–1106. doi: 10.1038/bmt.2017.14 28346417

[3] Castagnola E, Faraci M, Moroni C, Bandettini R, Caruso S, Bagnasco F, et al. Bacteremias in children receiving hemopoietic SCT. Bone Marrow Transplant. 2008; 41 Suppl 2: S104–106. doi: 10.1038/bmt.2008.66 18545230

[4] Sava M, Bättig V, Gerull S, Passweg JR, Khanna N, Garzoni C, et al. Bloodstream infections in allogeneic haematopoietic cell recipients from the Swiss Transplant Cohort Study: trends of causative pathogens and resistance rates. Bone Marrow Transplant. 2023; 58: 115–118. doi: 10.1038/s41409-022-01851-y 36310245 PMC9812769

[5] Mikulska M, Del Bono V, Raiola AM, Bruno B, Gualandi F, Occhini D, et al. Blood stream infections in allogeneic hematopoietic stem cell transplant recipients: reemergence of Gram-negative rods and increasing antibiotic resistance. Biol Blood Marrow Transplant. 2009; 15: 47–53. doi: 10.1016/j.bbmt.2008.10.024 19135942

[6] Youssef A, Hafez H, Madney Y, Elanany M, Hassanain O, Lehmann LE, et al. Incidence, risk factors, and outcome of blood stream infections during the first 100 days post-pediatric allogeneic and autologous hematopoietic stem cell transplantations. Pediatr Transplant. 2020; 24: e13610. doi: 10.1111/petr.13610 31682054

[7] Ustun C, Young JH, Papanicolaou GA, Kim S, Ahn KW, Chen M, et al. Bacterial blood stream infections (BSIs), particularly post-engraftment BSIs, are associated with increased mortality after allogeneic hematopoietic cell transplantation. Bone Marrow Transplant. 2019; 54: 1254–1265. doi: 10.1038/s41409-018-0401-4 30546070 PMC6565512

[8] Akhmedov M, Klyasova G, Kuzmina L, Fedorova A, Drokov M, Parovichnikova E. Post-engraftment Bloodstream Infections After Allogeneic Hematopoietic Cell Transplantation: Risk Factors and Association with Mortality. Infect Chemother. 2023; 55: 204–213. doi: 10.3947/ic.2022.0146 37038730 PMC10323536

[9] Mitchell AE, Derrington P, Turner P, Hunt LP, Oakhill A, Marks DI. Gram-negative bacteraemia (GNB) after 428 unrelated donor bone marrow transplants (UD-BMT): risk factors, prophylaxis, therapy and outcome. Bone Marrow Transplant. 2004; 33: 303–310. doi: 10.1038/sj.bmt.1704338 14647252

[10] Ballen K, Woo Ahn K, Chen M, Abdel-Azim H, Ahmed I, Aljurf M, et al. Infection Rates among Acute Leukemia Patients Receiving Alternative Donor Hematopoietic Cell Transplantation. Biol Blood Marrow Transplant. 2016; 22: 1636–1645. doi: 10.1016/j.bbmt.2016.06.012 27343716 PMC5008458

[11] Kim SH, Kee SY, Lee DG, Choi SM, Park SH, Kwon JC, et al. Infectious complications following allogeneic stem cell transplantation: reduced-intensity vs. myeloablative conditioning regimens. Transpl Infect Dis. 2013; 15: 49–59. doi: 10.1111/tid.12003 22998745

[12] Kikuchi M, Akahoshi Y, Nakano H, Ugai T, Wada H, Yamasaki R, et al. Risk factors for pre- and post-engraftment bloodstream infections after allogeneic hematopoietic stem cell transplantation. Transpl Infect Dis. 2015; 17: 56–65. doi: 10.1111/tid.12345 25580541

[13] Kanda Y. Investigation of the freely available easy-to-use software ’EZR’ for medical statistics. Bone Marrow Transplant. 2013; 48: 452–458. doi: 10.1038/bmt.2012.244 23208313 PMC3590441

[14] Akhmedov M, Klyasova G, Kuzmina L, Fedorova A, Vasilyeva V, Drokov M, et al. Incidence, etiology, risk factors, and outcomes of pre-engraftment bloodstream infections after first and second allogeneic hematopoietic cell transplantation. Transpl Infect Dis. 2022; 24: e13842. doi: 10.1111/tid.13842 35501664

[15] Bacigalupo A, Ballen K, Rizzo D, Giralt S, Lazarus H, Ho V, et al. Defining the intensity of conditioning regimens: working definitions. Biol Blood Marrow Transplant. 2009; 15: 1628–1633. doi: 10.1016/j.bbmt.2009.07.004 19896087 PMC2861656

[16] Park JR, Kreissman SG, London WB, Naranjo A, Cohn SL, Hogarty MD, et al. Effect of Tandem Autologous Stem Cell Transplant vs Single Transplant on Event-Free Survival in Patients With High-Risk Neuroblastoma: A Randomized Clinical Trial. JAMA. 2019; 322: 746–755. doi: 10.1001/jama.2019.11642 31454045 PMC6714031

[17] Pasqualini C, Dufour C, Goma G, Raquin MA, Lapierre V, Valteau-Couanet D. Tandem high-dose chemotherapy with thiotepa and busulfan-melphalan and autologous stem cell transplantation in very high-risk neuroblastoma patients. Bone Marrow Transplant. 2016; 51: 227–231. doi: 10.1038/bmt.2015.264 26524264

[18] Murthy HS, Gharaibeh RZ, Al-Mansour Z, Kozlov A, Trikha G, Newsome RC, et al. Baseline Gut Microbiota Composition Is Associated with Major Infections Early after Hematopoietic Cell Transplantation. Biol Blood Marrow Transplant. 2020; 26: 2001–2010. doi: 10.1016/j.bbmt.2020.07.023 32717434

[19] Badia P, Andersen H, Haslam D, Nelson AS, Pate AR, Golkari S, et al. Improving Oral Health and Modulating the Oral Microbiome to Reduce Bloodstream Infections from Oral Organisms in Pediatric and Young Adult Hematopoietic Stem Cell Transplantation Recipients: A Randomized Controlled Trial. Biol Blood Marrow Transplant. 2020; 26: 1704–1710. doi: 10.1016/j.bbmt.2020.05.019 32505810 PMC11168732

[20] Laheij AMGA, Raber-Durlacher JE, Koppelmans RGA, Huysmans MDNJ, Potting C, van Leeuwen SJM, et al. Microbial changes in relation to oral mucositis in autologous hematopoietic stem cell transplantation recipients. Sci Rep. 2019; 9: 16929. doi: 10.1038/s41598-019-53073-w 31729407 PMC6858439

[21] Laheij AMGA, Rozema FR, Brennan MT, von Bültzingslöwen I, van Leeuwen SJM, Potting C, et al. Long-Term Analysis of Resilience of the Oral Microbiome in Allogeneic Stem Cell Transplant Recipients. Microorganisms. 2022; 10. doi: 10.3390/microorganisms10040734 35456787 PMC9030553

[22] Lehrnbecher T, Fisher BT, Phillips B, Alexander S, Ammann RA, Beauchemin M, et al. Guideline for Antibacterial Prophylaxis Administration in Pediatric Cancer and Hematopoietic Stem Cell Transplantation. Clin Infect Dis. 2020; 71: 226–236. doi: 10.1093/cid/ciz1082 31676904 PMC7312235

[23] Ifversen M, Meisel R, Sedlacek P, Kalwak K, Sisinni L, Hutt D, et al. Supportive Care During Pediatric Hematopoietic Stem Cell Transplantation: Prevention of Infections. A Report From Workshops on Supportive Care of the Paediatric Diseases Working Party (PDWP) of the European Society for Blood and Marrow Transplantation (EBMT). Front Pediatr. 2021; 9: 705179. doi: 10.3389/fped.2021.705179 34395344 PMC8358428

[24] Lehrnbecher T, Averbuch D, Castagnola E, Cesaro S, Ammann RA, Garcia-Vidal C, et al. 8th European Conference on Infections in Leukaemia: 2020 guidelines for the use of antibiotics in paediatric patients with cancer or post-haematopoietic cell transplantation. Lancet Oncol. 2021; 22: e270–e280. doi: 10.1016/S1470-2045(20)30725-7 33811814

[25] Gardner JC, Courter JD, Dandoy CE, Davies SM, Teusink-Cross A. Safety and Efficacy of Prophylactic Levofloxacin in Pediatric and Adult Hematopoietic Stem Cell Transplantation Patients. Transplant Cell Ther. 2022; 28: 167.e161–167.e165. doi: 10.1016/j.jtct.2021.11.017 34875405

[26] Noel GJ, Bradley JS, Kauffman RE, Duffy CM, Gerbino PG, Arguedas A, et al. Comparative safety profile of levofloxacin in 2523 children with a focus on four specific musculoskeletal disorders. Pediatr Infect Dis J. 2007; 26: 879–891. doi: 10.1097/INF.0b013e3180cbd382 17901792

[27] Alrugaib T, Alsultan A, Elbashir E, Albdah B, Alharbi M, Essa MF. Antimicrobial prophylaxis and the rate of blood stream infections and Clostridioides difficile in pediatric stem cell transplantation: A single-center retrospective study. Pediatr Transplant. 2023; 27: e14375. doi: 10.1111/petr.14375 35946349

[28] Jaffray J, Witmer C, O’Brien SH, Diaz R, Ji L, Krava E, et al. Peripherally inserted central catheters lead to a high risk of venous thromboembolism in children. Blood. 2020; 135: 220–226. doi: 10.1182/blood.2019002260 31909784

[29] Ullman AJ, Marsh N, Mihala G, Cooke M, Rickard CM. Complications of Central Venous Access Devices: A Systematic Review. Pediatrics. 2015; 136: e1331–1344. doi: 10.1542/peds.2015-1507 26459655

[30] Miyagishima M, Hamada M, Hirayama Y, Muramatsu H, Tainaka T, Shirota C, et al. Risk factors for unplanned removal of central venous catheters in hospitalized children with hematological and oncological disorders. Int J Hematol. 2022; 116: 288–294. doi: 10.1007/s12185-022-03346-4 35727532

[31] Annetta MG, Celentano D, Zumstein L, Attinà G, Ruggiero A, Conti G, et al. Catheter-related complications in onco-hematologic children: A retrospective clinical study on 227 central venous access devices. J Vasc Access. 2022: 11297298221122128. doi: 10.1177/11297298221122128 36113076

[32] Elgarten CW, Otto WR, Shenton L, Stein MT, Horowitz J, Aftandilian C, et al. Risk of bacterial bloodstream infection does not vary by central-line type during neutropenic periods in pediatric acute myeloid leukemia. Infect Control Hosp Epidemiol. 2023; 44: 222–229. doi: 10.1017/ice.2022.82 35465865

