# Supplementary figures and images for: Risk factors of bloodstream infection after allogeneic hematopoietic cell transplantation in children/adolescent and young adults
Source: PLoS One. 2024 Aug 7;19(8):e0308395. doi: 10.1371/journal.pone.0308395 (PMC11305574; doi:10.1371/journal.pone.0308395)

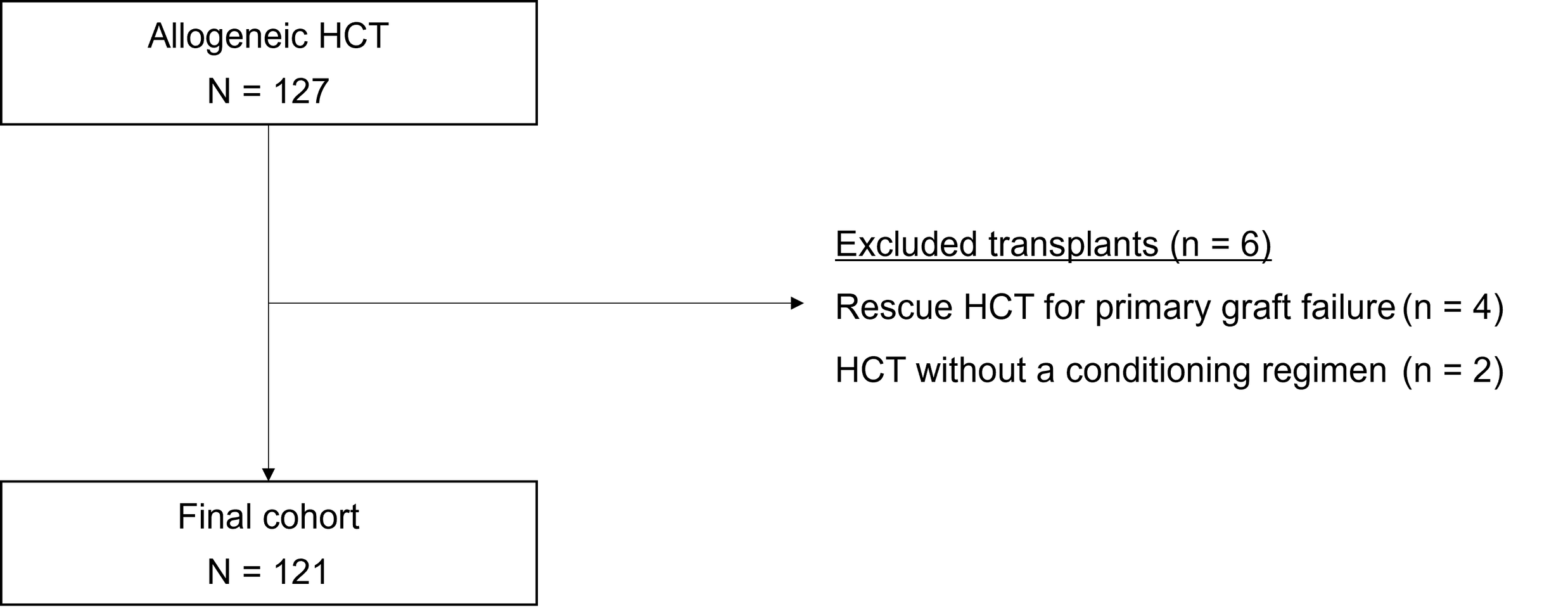

Supplement: S1 Fig — There were 127 allogeneic HCTs. Six HCTs were excluded, and the final cohort included 121 HCTs. Abbreviations: HCT, hematopoietic cell transplantation. (TIF) [file pone.0308395.s001.tif]

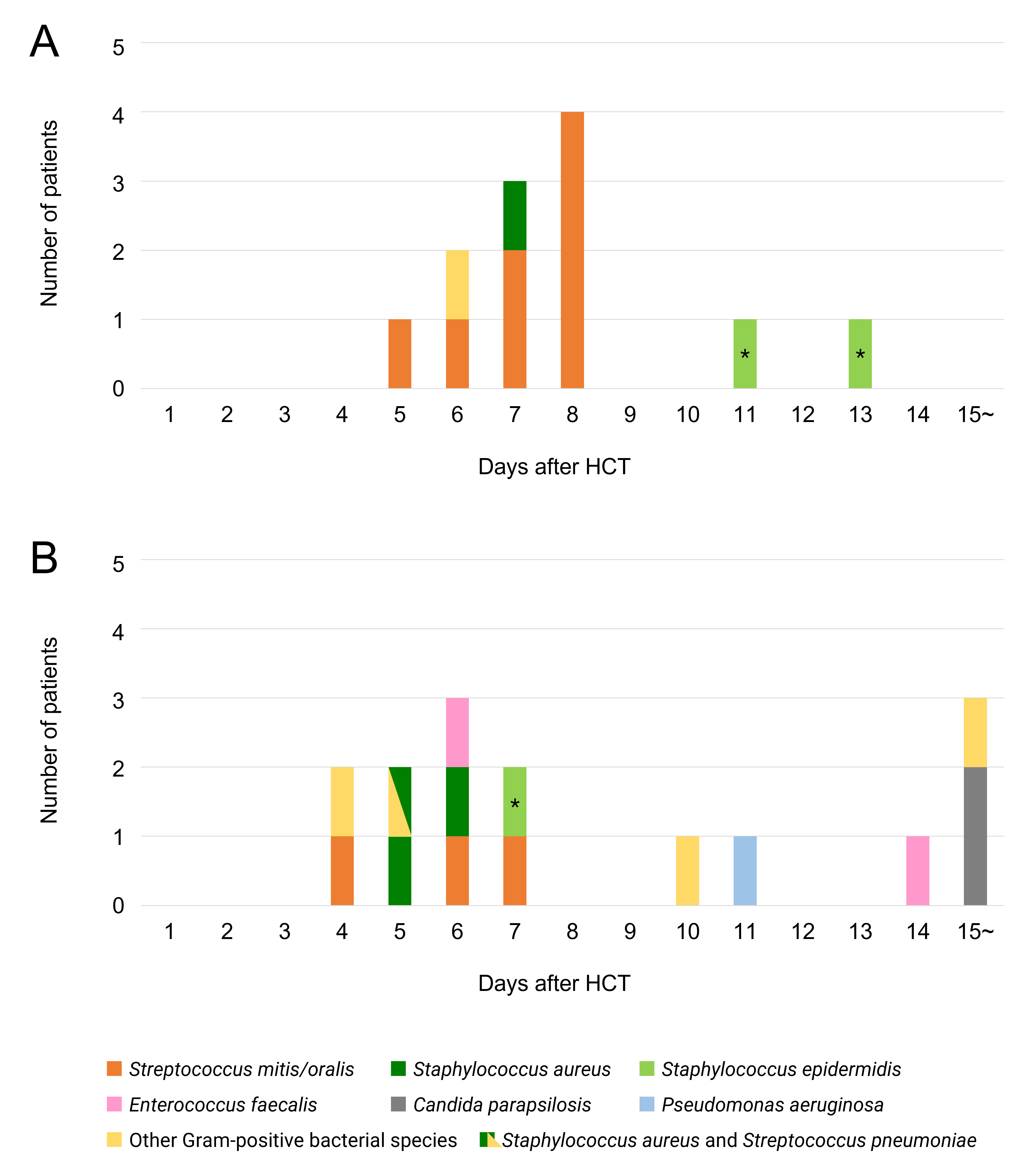

Supplement: S2 Fig — The number of patients based on the onset date of bloodstream infection in patients who received (A) tandem HCT and (B) non-tandem HCT is indicated by a different color for each pathogen isolated. The asterisk (*) indicates probable BSI patients. Abbreviations: HCT, hematopoietic cell transplantation. (TIF) [file pone.0308395.s002.tif]
