# Supplementary material for: Risk factors of bloodstream infection after allogeneic hematopoietic cell transplantation in children/adolescent and young adults
Source: PLoS One. 2024 Aug 7;19(8):e0308395. doi: 10.1371/journal.pone.0308395 (PMC11305574; doi:10.1371/journal.pone.0308395)
Supplement: S2 Table — (DOCX) [file pone.0308395.s004.docx]

**Supplemental Table 2. Pathogens isolated from blood culture in patients with tandem and non-tandem HCT**

| Species | | All patients  (n = 121) | Tandem HCT  (n = 21) | Non-tandem HCT  (n = 100) |
| --- | --- | --- | --- | --- |
| **Number of blood stream infections, n** | | **27** | **12** | **15** |
| **Gram-positive bacteria, n (%)** | | **25 (89)** | **12 (100)** | **13 (81)** |
|  | *Streptococcus mitis/oralis*, n | 11 | 8 | 3 |
|  | *Staphylococcus aureus*, n | 4* | 1 | 3* |
|  | *Staphylococcus epidermidis*, n | 3** | 2** | 1** |
|  | *Enterococcus faecalis*, n | 2 | 0 | 2 |
|  | *Enterococcus faecium*, n | 1 | 0 | 1 |
|  | *Gemella haemolysans*, n | 1 | 0 | 1 |
|  | *Staphylococcus haemolyticus*, n | 1 | 0 | 1 |
|  | *Streptococcus agalactiae*, n | 1 | 1 | 0 |
|  | *Streptococcus pneumoniae*, n | 1* | 0 | 1* |
| **Gram-negative bacteria, n (%)** | | **1 (4)** | **0 (0)** | **1 (6)** |
|  | *Pseudomonas aeruginosa*, n | 1 | 0 | 1 |
| **Fungus, n (%)** | | **2 (7)** | **0 (0)** | **2 (13)** |
|  | *Candida parapsilosis*, n | 2 | 0 | 2 |

*In one patient, *Staphylococcus aureus* and *Streptococcus pneumoniae* were simultaneously isolated from blood culture in a single blood stream infection episode. **Probable BSI (2 patients in tandem HCT and 1 patient in non-tandem HCT).

CVC, central venous catheter; HCT, hematopoietic cell transplantation; PICC, peripherally inserted central catheter
