# Supplementary material for: Risk factors of bloodstream infection after allogeneic hematopoietic cell transplantation in children/adolescent and young adults
Source: PLoS One. 2024 Aug 7;19(8):e0308395. doi: 10.1371/journal.pone.0308395 (PMC11305574; doi:10.1371/journal.pone.0308395)
Supplement: S3 Table — (DOCX) [file pone.0308395.s005.docx]

**Supplemental Table 3. Patient and transplantation characteristics of tandem HCT**

|  | Tandem HCT patients | Patients with  BSI* | Patients without BSI |
| --- | --- | --- | --- |
|  | N = 21 | n = 12 | n = 9 |
| Age at HCT, n (%) |  |  |  |
| < 6 years old | 14 (67) | 7 (58) | 7 (78) |
| ≥ 6 years old | 7 (33) | 5 (42) | 2 (22) |
| Gender, n (%) |  |  |  |
| Male | 11 (52) | 7 (58) | 4 (44) |
| Female | 10 (48) | 5 (42) | 5 (56) |
| Disease, n (%) |  |  |  |
| Benign diseases | 0 (0) | 0 (0) | 0 (0) |
| Hematological malignancies | 0 (0) | 0 (0) | 0 (0) |
| Solid tumors | 21 (100) | 12 (100) | 9 (100) |
| Donor type, n (%) |  |  |  |
| Matched related (8/8 allele matched) | 0 (0) | 0 (0) | 0 (0) |
| Mismatched related (7/8 allele matched) | 0 (0) | 0 (0) | 0 (0) |
| Matched unrelated (8/8 allele matched) | 0 (0) | 0 (0) | 0 (0) |
| Mismatched unrelated (7/8 allele matched) | 0 (0) | 0 (0) | 0 (0) |
| Haploidentical | 0 (0) | 0 (0) | 0 (0) |
| Cord blood | 21 (100) | 12 (100) | 9 (100) |
| Conditioning regimen, n (%) |  |  |  |
| Myeloablative conditioning | 0 (0) | 0 (0) | 0 (0) |
| Reduced intensity conditioning | 21 (100) | 12 (100) | 9 (100) |
| Total body irradiation, n (%) |  |  |  |
| None | 0 (0) | 0 (0) | 0 (0) |
| Low dose (< 8 Gy) | 21 (100) | 12 (100) | 9 (100) |
| High dose (≥ 8 Gy) | 0 (0) | 0 (0) | 0 (0) |
| Catheter, n (%) |  |  |  |
| Tunneled CVC | 20 (95) | 11 (92) | 9 (100) |
| PICC | 1 (5) | 1 (8) | 0 (0) |
| Catheter retention time, n (%) |  |  |  |
| < 45 days | 3 (14) | 1 (8) | 2 (22) |
| ≥ 45 days | 18 (86) | 11 (92) | 7 (78) |
| Antibiotic use at day 0 of HCT, n (%) |  |  |  |
| No | 19 (90) | 11 (92) | 8 (89) |
| Yes | 2 (10) | 1 (8) | 1 (11) |
| History of BSI within 6 months prior to HCT, n (%) |  |  |  |
| No | 20 (95) | 12 (100) | 8 (89) |
| Yes | 1 (5) | 0 (0) | 1 (11) |
| Active infections at the time of HCT, n (%) |  |  |  |
| No | 21 (100) | 12 (100) | 9 (100) |
| Yes | 0 (0) | 0 (0) | 0 (0) |
| Oral mucositis (CTCAE v5.0) |  |  |  |
| < Grade 2 | 16 (76) | 9 (75) | 7 (78) |
| ≥ Grade 2 | 5 (24) | 3 (25) | 2 (22) |

*Definitive BSI (n = 10) and probable BSI (n = 2). Abbreviations: BSI, bloodstream infection; CTCAE, common terminology criteria for adverse events; CVC, central venous catheter; HCT, hematopoietic cell transplantation; HLA, human leukocyte antigen; PICC, peripherally inserted central catheter.
