# Supplementary material for: Risk factors of bloodstream infection after allogeneic hematopoietic cell transplantation in children/adolescent and young adults
Source: PLoS One. 2024 Aug 7;19(8):e0308395. doi: 10.1371/journal.pone.0308395 (PMC11305574; doi:10.1371/journal.pone.0308395)
Supplement: S4 Table — (DOCX) [file pone.0308395.s006.docx]

**Supplemental Table 4. Univariable analyses of cumulative incidence of blood stream infections in tandem HCT patients**

| Variable | Univariable | |
| --- | --- | --- |
|  | HR (95% CI) | *P* |
| Age at HCT |  |  |
| < 6 years old | 1 |  |
| ≥ 6 years old | 1.25 (0.48–3.25) | 0.650 |
| Gender |  |  |
| Male | 1 |  |
| Female | 0.79 (0.28–2.26) | 0.660 |
| Catheter |  |  |
| Tunneled CVC | 1 |  |
| PICC | 1.54 (0.86–2.75) | 0.150 |
| Catheter retention time |  |  |
| < 45 days | 1 |  |
| ≥ 45 days | 2.50 (0.46–13.5) | 0.290 |
| Antibiotic use at day 0 of HCT |  |  |
| No | 1 |  |
| Yes | 0.66 (0.14–3.04) | 0.600 |
| Oral mucositis (CTCAE v5.0) |  |  |
| < Grade 2 | 1 |  |
| ≥ Grade 2 | 0.19 (0.03–1.05) | 0.056 |

BM, bone marrow; CB, cord blood; CI, confidence intervals; CTCAE, common terminology criteria for adverse events; CVC, central venous catheter; HCT, hematopoietic cell transplantation; HLA, human leukocyte antigen; HR, hazard ratio; PBSC, peripheral blood stem cells; PICC, peripherally inserted central catheter.
