# Supplementary material for: Risk factors of bloodstream infection after allogeneic hematopoietic cell transplantation in children/adolescent and young adults
Source: PLoS One. 2024 Aug 7;19(8):e0308395. doi: 10.1371/journal.pone.0308395 (PMC11305574; doi:10.1371/journal.pone.0308395)
Supplement: S5 Table — (DOCX) [file pone.0308395.s007.docx]

**Supplemental Table 5. Patient and transplantation characteristics of non-tandem HCT**

|  | Non-tandem HCT patients | Patients with  BSI* | Patients without BSI |
| --- | --- | --- | --- |
|  | N = 100 | n = 15 | n = 85 |
| Age at HCT, n (%) |  |  |  |
| < 6 years old | 40 (40) | 5 (33) | 35 (41) |
| ≥ 6 years old | 60 (60) | 10 (67) | 50 (59) |
| Gender, n (%) |  |  |  |
| Male | 61 (61) | 5 (33) | 56 (66) |
| Female | 39 (39) | 10 (67) | 29 (34) |
| Disease, n (%) |  |  |  |
| Benign diseases | 42 (42) | 5 (33) | 37 (44) |
| Hematological malignancies | 35 (35) | 2 (13) | 33 (39) |
| Solid tumors | 23 (23) | 8 (53) | 15 (18) |
| Donor type, n (%) |  |  |  |
| Matched related (8/8 allele matched) | 13 (13) | 0 (0) | 13 (15) |
| Mismatched related (7/8 allele matched) | 3 (3) | 0 (0) | 3 (4) |
| Matched unrelated (8/8 allele matched) | 15 (15) | 1 (7) | 14 (16) |
| Mismatched unrelated (7/8 allele matched) | 20 (20) | 4 (27) | 16 (19) |
| Haploidentical | 11 (11) | 1 (7) | 10 (12) |
| Cord blood | 38 (38) | 9 (60) | 31 (36) |
| Conditioning regimen, n (%) |  |  |  |
| Myeloablative conditioning | 49 (49) | 4 (27) | 45 (53) |
| Reduced intensity conditioning | 51 (51) | 11 (73) | 40 (47) |
| Total body irradiation, n (%) |  |  |  |
| None | 19 (19) | 0 (0) | 19 (22) |
| Low dose (< 8 Gy) | 49 (49) | 11 (73) | 38 (45) |
| High dose (≥ 8 Gy) | 32 (32) | 4 (27) | 28 (33) |
| Catheter, n (%) |  |  |  |
| Tunneled CVC | 81 (81) | 9 (60) | 72 (85) |
| PICC | 17 (17) | 6 (40) | 11 (13) |
| Tunneled CVC and PICC | 2 (2) | 0 (0) | 2 (2) |
| Catheter retention time, n (%) |  |  |  |
| < 45 days | 58 (58) | 7 (47) | 51 (60) |
| ≥ 45 days | 42 (42) | 8 (53) | 34 (40) |
| Antibiotic use at day 0 of HCT, n (%) |  |  |  |
| No | 48 (48) | 8 (53) | 40 (47) |
| Yes | 52 (52) | 7 (47) | 45 (53) |
| History of BSI within 6 months prior to HCT, n (%) |  |  |  |
| No | 86 (86) | 12 (80) | 74 (87) |
| Yes | 14 (14) | 3 (20) | 11 (13) |
| Active infections at the time of HCT, n (%) |  |  |  |
| No | 94 (94) | 14 (93) | 80 (94) |
| Yes | 6 (6) | 1 (7) | 5 (6) |
| Oral mucositis (CTCAE v5.0) |  |  |  |
| < Grade 2 | 47 (47) | 7 (47) | 40 (47) |
| ≥ Grade 2 | 53 (53) | 8 (53) | 45 (53) |

*Definitive BSI (n = 14) and probable BSI (n = 1). Abbreviations: BSI, bloodstream infection; CTCAE, common terminology criteria for adverse events; CVC, central venous catheter; HCT, hematopoietic cell transplantation; HLA, human leukocyte antigen; PICC, peripherally inserted central catheter.
