# Supplementary material for: Risk factors of bloodstream infection after allogeneic hematopoietic cell transplantation in children/adolescent and young adults
Source: PLoS One. 2024 Aug 7;19(8):e0308395. doi: 10.1371/journal.pone.0308395 (PMC11305574; doi:10.1371/journal.pone.0308395)
Supplement: S6 Table — (DOCX) [file pone.0308395.s008.docx]

**Supplemental Table 6. Univariable analyses of cumulative incidence of blood stream infections in non-tandem HCT patients**

| Variable | Univariable | |
| --- | --- | --- |
|  | HR (95% CI) | *P* |
| Age at HCT |  |  |
| < 6 years old | 1 |  |
| ≥ 6 years old | 1.39 (0.48–3.99) | 0.540 |
| Gender |  |  |
| Male | 1 |  |
| Female | 3.56 (1.25–10.1) | 0.017 |
| Disease |  |  |
| Benign | 1 |  |
| Malignant | 1.57 (0.56–4.44) | 0.390 |
| Source |  |  |
| BM and/or PBSC | 1 |  |
| CB | 2.72 (0.99–7.47) | 0.053 |
| HLA (8 allele) |  |  |
| Matched | 1 |  |
| Mismatched | 6.22 (0.83–46.7) | 0.075 |
| Conditioning regimen |  |  |
| Myeloablative conditioning | 1 |  |
| Reduced intensity conditioning | 2.76 (0.88–8.67) | 0.083 |
| Total body irradiation |  |  |
| < 8 Gy | 1 |  |
| ≥ 8 Gy | 0.78 (0.25–2.45) | 0.670 |
| Catheter |  |  |
| Tunneled CVC | 1 |  |
| PICC | 3.51 (1.29–9.58) | 0.014 |
| Catheter retention time |  |  |
| < 45 days | 1 |  |
| ≥ 45 days | 1.70 (0.63–4.60) | 0.300 |
| Antibiotic use at day 0 of HCT |  |  |
| No | 1 |  |
| Yes | 0.75 (0.28–2.05) | 0.580 |
| History of BSI within 6 months prior to HCT |  |  |
| No | 1 |  |
| Yes | 1.48 (0.45–4.81) | 0.520 |
| Active infections at the time of HCT |  |  |
| No | 1 |  |
| Yes | 1.10 (0.16–7.86) | 0.920 |
| Oral mucositis (CTCAE v5.0) |  |  |
| < Grade 2 | 1 |  |
| ≥ Grade 2 | 1.04 (0.39–2.82) | 0.940 |

BM, bone marrow; BSI, bloodstream infection; CB, cord blood; CI, confidence intervals; CTCAE, common terminology criteria for adverse events; CVC, central venous catheter; HCT, hematopoietic cell transplantation; HLA, human leukocyte antigen; HR, hazard ratio; PBSC, peripheral blood stem cells; PICC, peripherally inserted central catheter.
